# Culturally competent communication in Indigenous disability assessment: a qualitative study

**DOI:** 10.1186/s12939-021-01402-9

**Published:** 2021-03-01

**Authors:** Angeline Ferdinand, Libby Massey, Jennifer Cullen, Jeromey Temple, Kristy Meiselbach, Yin Paradies, Gareth Baynam, Ravi Savarirayan, Margaret Kelaher

**Affiliations:** 1 Centre for Health Policy, School of Population and Global Health, University of Melbourne, Parkville, VIC 3011 Australia; 2 MJD Foundation, Nightcliff, NT 0814 Australia; 3 James Cook University, Cairns,, QLD 4870 Australia; 4 Synapse, Level 1/262 Montague Road, West End, QLD 4101 Australia; 5 Alfred Deakin Institute for Citizenship and Globalisation, Deakin University, 221 Burwood Highway, Burwood, VIC 3125 Australia; 6 Western Australian Register of Developmental Anomalies and Genetic Services of Western Australia, King Edward Memorial Hospital, 374 Bagot Rd, Subiaco, WA 6008 Australia; 7 Telethon Kids Institute and the Faculty of Health and Medical Science, Division of Paediatrics, University of Western Australia, Monash Avenue, Nedlands, WA 6009 Australia; 8 Victorian Clinical Genetics Services, The Royal Children’s Hospital, 50 Flemington Rd, Parkville, VIC 3052 Australia; 9 University of Melbourne, Parkville, VIC 3052 Australia

**Keywords:** Indigenous health, Disability, Cultural competence, Australia

## Abstract

**Background:**

Indigenous people tend to exhibit a higher burden of disability than their non-Indigenous counterparts, and are often underserved by disability services. Engaging appropriately with Indigenous communities, families and individuals in the initial stages of disability assessment and planning is crucial in order to build trust and understanding of disability service models and ensure that Indigenous people receive support that is tailored to their needs and cultural realities. This article aims to identify key elements of culturally competent communication in Indigenous disability assessment and planning, and provide recommendations for strengthening capacity in this area.

**Methods:**

This qualitative research was designed to involve Aboriginal and Torres Strait Islander people at all stages and to reflect the views of Aboriginal and Torres Strait Islander researchers, people and families affected by disability and the community-controlled health sector. Semi-structured individual interviews were undertaken with staff implementing the National Disability Insurance Scheme (NDIS) (*n* = 4), NDIS participants (*n* = 24), disability support providers and organisational partners (*n* = 19) and Community Connectors (*n* = 8) in Queensland and the Northern Territory of Australia. Key themes derived from thematic analysis included appropriate and adequate engagement of individuals with disability and their families, the role of trusted relationships, and culturally safe and appropriate communication during planning meetings.

**Results:**

Overall, the research findings highlight that a low level of cultural competence in the initial stages of the disability assessment and planning process exacerbated participant confusion and distrust towards assessment staff and the NDIS. Given difficulties in communication, participant understanding of the NDIS was generally limited. The necessity of culturally safe and appropriate use of interpreters was stressed, as was the role of trusted individuals, including existing service providers, Community Connectors and family members in providing a solid base for participant understanding of the NDIS.

**Conclusions:**

Cultural competence in disability assessment and planning can be strengthened through multi-level engagement with the Aboriginal community-controlled sector and community leaders. Implementing mechanisms to enable the involvement of families, trusted service providers and Community Connectors can support a more meaningful understanding of individuals’ needs within their cultural context and in relation to their cultural roles.

**Supplementary Information:**

The online version contains supplementary material available at 10.1186/s12939-021-01402-9.

## Background

Available evidence indicates that Indigenous peoples tend to have higher levels of disability than non-Indigenous people in the same country. Aboriginal and Torres Strait Islanders have been found to be nearly twice as likely to experience disability than non-Indigenous people [1–3]. In Canada, Indigenous people were more likely to have at least one disability compared to non-Indigenous people. This difference was especially marked for Indigenous women, who were also more likely to experience severe or very severe disabilities than non-Indigenous women [4, 5]. In New Zealand, Māori have higher proportions of disability than non-Māori across all age groups [6]. Despite being more strongly affected by disability in comparison to non-Indigenous populations, Indigenous people are neglected in disability research and literature [2, 4]. Limited understanding and consideration of how Indigenous people experience disability may hinder the development of accessible services and the creation of disability policy that is appropriately responsive to the needs and realities of Indigenous people with disabilities [2, 3].

There has been a recognition that one-size-fits-all models of disability care are less effective than those that allow individuals to choose their services and provide individualised support [7, 8]. This has led to the rise in individualised models of funding across a number of high-income countries including Australia, Canada, and some European countries [9]. Personalised funding models aim to support the empowerment of people with disabilities to articulate and work towards their own identified goals and aspirations as well as increase accountability in service provision and flexibility in how needs are met [9, 10]. However, Indigenous people may not receive the full benefits of personalised funding programs without adequate mechanisms in place to ensure incorporation of Indigenous understandings of disability.

Procedures for assessing eligibility have been identified as a potential barrier to accessing disability support [11, 12]. The assessment process can be complex and subjective, involving review of not only impairments, but how such impairments affect functioning in daily activities. Shared understanding between individuals, families and assessors is therefore necessary, but may be complicated by cultural factors. Indigenous peoples’ understanding of disability and health can be markedly different than that of non-Indigenous people. In many Indigenous language groups, there is no equivalent word for ‘disability’ or for specific disabilities [13–17], and many Indigenous people living with disabilities do not self-identify as having a disability [13, 18]. The language of disability and under-identification of disability can therefore serve as a barrier [18], limiting engagement with services specific to ‘disabilities’ and contributing to under-reporting and under-utilisation of disability services [14, 19]. Additionally, research has indicated marked differences between needs identified by Indigenous people with disability and the supports mainstream services are designed to provide [20]. Engaging appropriately with Indigenous communities, families and people in the initial stages of implementing a new disability scheme is crucial in order to build trust and understanding of the scheme, enabling individuals and their families to go through the enrolment process, and ensuring that Indigenous people are adequately represented and that they receive support that is tailored to their needs.

Given the importance of this initial phase, appropriate communication between individuals with disability and assessors is crucial. Developing cultural competence is a key strategy that has been utilised to address inequities in access to health care services and improve the quality of health care services for Indigenous people [21]. Cultural competence, also referred to as cultural safety, has been defined as ‘a set of congruent behaviours, attitudes and policies that come together in a system, agency or among professionals; enabling that system, agency or those professionals to work effectively in cross-cultural situations [22].’ Cultural competence refers not only to interpersonal communication skills, but rather encompasses institutional and community-level practice [21]. While often referring to inter-racial or inter-ethnic interactions, cultural competence frameworks have less frequently been applied to examine health care provision to people with disability [23, 24]. However, a 2016 review of the literature on cultural competence in service provision to people with disability indicated a lack of focus on the intersection between disability and racial/ethnic identity [24].

Described as the largest reform of the Australian disability services sector [19, 25, 26], the National Disability Insurance Scheme (NDIS) was introduced in 2013 by the Federal Government of Australia. Managed by the National Disability Insurance Agency (NDIA), the NDIS was initially trialled in selected communities in 2013, with a gradual national roll-out to all communities in Australia beginning in 2016 [25]. The purpose of the reform was to provide tailored, personalised funding packages for disability supports to Australians living with permanent and significant disability [25]. People are eligible for the NDIS if they are an Australian citizen, permanent resident or permanent visa holder, are under 65 years of age when they enter the scheme and have a permanent (lifelong) and significant disability. An estimated 60,000 Aboriginal and Torres Strait Islander people nationally are eligible for participation in the NDIS [27]. This qualitative research focuses on the experiences of Aboriginal participants in the initial stages of engaging with the NDIS and NDIA staff in the Northern Territory and Queensland. The research takes a case study approach to identify key elements of culturally competent communication in Indigenous disability assessment and planning, and provide recommendations for strengthening capacity in this area.

## Methods

### Governance

The research was conducted in collaboration with the Machado-Joseph Disease (MJD) Foundation (MJDF) and Synapse. These organisations have longstanding connections with Aboriginal and Torres Strait Islander communities in the Northern Territory and Queensland respectively. The project built on these strong relationships with Aboriginal and Torres Strait Islander communities, which enabled intimate access to participants, resource sharing and the expertise of highly experienced disability and community service professionals across design, planning and data gathering phases of the project. Synapse are committed to reducing the disadvantage that Aboriginal and Torres Strait Islander people living with disability face and are working to connect more Aboriginal and Torres Strait Islander people with the NDIS. MJDF is a charity founded in 2008 which works in partnership with Aboriginal Australians, their families and communities to support those living with the genetic neurodegenerative condition Machado-Joseph disease (MJD).

The project was designed to involve Aboriginal and Torres Strait Islander people at all stages, leading project development, implementation and dissemination. The project aimed to ensure it reflected the views of Aboriginal and Torres Strait Islander researchers, people and families affected by disability and the community-controlled health sector. This was achieved by recruiting a research team where four of the nine members were Aboriginal researchers. In addition to the research team, Aboriginal leadership was also an important aspect of the Project Reference Group (PRG), where five of the thirteen members were Aboriginal. Aboriginal and Torres Strait Islander people living with disabilities participated in all stages of the project, including the PRG, co-design process, data collection and analysis.

This project was approved by the Human Research Ethics Committee for the Northern Territory Department of Health and the Menzies School of Health Research (HREC 2018–3175).

### Sample

Sites for the project included Cairns, Townsville and four East Arnhem communities. Cairns and Townsville are small regional coastal cities in far north and north Queensland, with populations of approximately 150,000 and 230,000 respectively. In 2016, the Australian Census indicated that Aboriginal and/or Torres Strait Islander people are 9% of the population in Cairns and 8% in Townsville [28, 29]. East Arnhem comprises nine major remote communities in the north of the Northern Territory, with a population of approximately 9000. Over 90% of residents in East Arnhem are Aboriginal and/or Torres Strait Islander [30].

Senior NDIA staff were identified through the PRG and invited to participate in the research. Disability support providers registered with the NDIA and NDIA partner organisations were identified with the assistance of the PRG and project partners, as well as through the NDIA website. These individuals were initially approached by e-mail or telephone. While no potential NDIA interviewees declined to participate in the research directly, some reluctance was expressed due to perceptions that the NDIS had been facing a high amount of scrutiny. Moreover, a high level of turnover made it difficult in some cases to locate NDIA staff that had an appropriate level of experience in their role. A small number of NDIS participants declined to be interviewed; reasons included dissatisfaction with the NDIS and reluctance to be involved in research relating to the NDIS while these issues were outstanding. All NDIA staff, support providers and NDIA partner organisations who consented were interviewed. Community Connectors and participants were identified through the networks of the project partners and Aboriginal community interviewers and approached to participate in the research either face-to-face or via telephone. Community Connector and participant interviews were constrained by a season of cyclones and flooding, where several communities had to be evacuated. Sampling continued in unaffected communities until it was no longer feasible, at which point data saturation was also reached (Table 1).

**Table 1:** Interviewees’ roles and gender by jurisdiction

QLD		
	n	Gender
NDIA	1	1 Male
Participants	5	2 Males, 3 Females
Disability support providers	14	2 Males, 12 Females
Organisational partners	1	1 Male
NT		
NDIA	3	3 Females
Participants	19	3 Males, 16 Females
Disability support providers	3	1 Male, 2 Females
Organisational partners	1	1 Male
Community connectors	8	2 Males, 6 Females

### Interviews

Interview schedules were developed by the authors, with significant input into the process by MJDF and Synapse staff to ensure that the schedules utilised appropriate terminology and, for participant interview schedules, were culturally appropriate and understandable (see Supplementary Material for interview schedules). Participant interview schedules reflected the different steps taken by participants to obtain access to the NDIS, develop a participant plan and access supports. Interviews with NDIA staff, providers and partner organisations covered various aspects of engaging with community, NDIS funding arrangements, the process of registering providers, developing participant plans and providing culturally appropriate supports. Partner organisations are organisations that do not provide direct disability supports, but may provide support coordination, employ Community Connectors or collaborate with the NDIA in other ways. Community Connector interviews focused on community and participant engagement, the training provided by the NDIS, developing participant plans and understanding the NDIS.

Interviews were conducted between November 2018 and April 2019. Interviews with NDIA staff and organisational partners were undertaken by University of Melbourne research team members (AF). Support provider interviews were undertaken by University of Melbourne staff in the Northern Territory (AF) and a combination of University of Melbourne (AF) and Synapse staff (JC) in Queensland. Interviews with participants and Community Connectors were undertaken either by MJDF staff (LM) or Aboriginal and non-Aboriginal community researchers (not listed) with experience in providing disability support, and who spoke the relevant language of interviewees.

 Community researchers were male; all other interviewers were female. The University of Melbourne staff member is formally trained in qualitative research methods and research ethics, with extensive experience conducting qualitative research. This staff member conducted training in research ethics and qualitative data collection with the community researchers and MJDF staff. The University of Melbourne researcher did not have an established relationship with interviewees prior to the study. In contrast, MJDF and Synapse staff and community researchers were familiar with the individuals they interviewed prior to the study in their role as support providers; or, they were referred to research participants by known individuals.

All interviewees were informed of the purpose of the research and what it would entail. NDIA staff, providers, staff of partner organisations and Community Connectors received a Plain Language Statement regarding the research and returned a signed consent form. The majority of NDIS participants interviewed also provided a written consent form; however, there were some cases where that was not possible and verbal consent was provided instead. Interviews with NDIA staff, service providers and staff of partner organisations lasted approximately 45 minutes to 1 hour. NDIA staff and partner organisations and the majority of support providers were interviewed over the phone, with a minority of support providers interviewed face-to-face. Interviews with NDIS participants and Community Connectors were conducted face-to-face. Participants were invited to have another person present during the interview for support; in several cases, participants had family members present. Interviews with Community Connectors and participants varied between 15 minutes and 1 hour 15 min. Community Connectors and participants were provided a $50 grocery voucher following the interview to thank them for their involvement in the research. This amount was decided through consultation with the research partners and the community interviewers. Interviews were audio-recorded where consented to by interviewees . Where interviews were audio-recorded, they were then transcribed shortly after the interview, otherwise notes of the interview were provided by the interviewer. All interviewees were offered the opportunity to review transcripts before analysis; however, the majority of interviewees declined to review the transcripts. Audio and transcript data and interview notes were stored on password-protected University of Melbourne networks and were only accessible by University of Melbourne research team members. All identifiable information was removed from data prior to its use in any reporting. To ensure data integrity and enhance analysis, all transcriptions were cross-checked by the research team. Both transcripts and notes were entered into NVivo and coded for thematic analysis by a single coder (author AF), with themes being derived from the data. Following initial analysis, workshops were held with the PRG and project partners in April 2019 to ensure validity of the findings and refine the recommendations.

## Results

Overall, the research findings highlight a low level of cultural competence in initial NDIA interactions with NDIS participants and their families, which exacerbated participant confusion and distrust towards NDIA staff and the NDIS. Given difficulties in communication, participant understanding of the NDIS was generally limited, which would likely have negative implications for planning meetings to accurately reflect participant support needs. Key themes included the need for appropriate and adequate participant engagement, which interviewees considered to include prior participant preparation for the arrival of NDIA staff, in conjunction with trusted disability support workers and Community Connectors. Provisions for culturally safe and appropriate use of interpreters was often discussed during interviews, as was the role of Community Connectors in providing a solid base for participant understanding of the NDIS. The success of planning meetings in accurately reflecting the needs of participants as embedded in their cultural context and their experiences of disability was seen to be dependent on having each of these pieces in place.

### Participant experiences of disability

Overall, family and community was a strong theme in terms of the participants’ experiences of disabilities, their aspirations, their current care and the supports that they felt were necessary. The following quotes illustrate participants experiencing belonging and respect in the community as being protective against discrimination.

*I haven’t ever experienced other people being mean to me because of my disability. Everyone knows who I am and my family and clan, so that hasn’t happened.* (Participant).

***Interviewer:*** *Do you feel like you have been treated fair by other people, or unfair by other people in the … community? Are they treating you in the right way?*

***Participant:*** *Yeah, they treat me alright.*

***Interviewer:*** *Anyone ever been nasty to you because of this problem?*

***Participant:*** *Nah – cos they respect me there.*

Family was also a central concept as participants reflected on their own roles as parents or caregivers, as well as speaking about their family members as their primary carers. In some cases where the disease is both genetic and degenerative, like Machado-Joseph disease, capturing personal and family stories was also critically important to participants:

*I want to write my (MJD) story for my kids. I need to write it down now, before I lose my memory and before my voice fades away.* (Participant).

*It makes it hard for me to be a Dad, to be in my day … things like cooking, hunting, fishing, cleaning …* (Participant).

*My sister, she helps me get up go toilet make me have a shower, even L helps me too. They make my tea and when we go shopping I go and look – ‘I want that that, that’ and they are my arms and legs. My nieces help push me in the chair.* (Participant).

*The other important thing is respite, staying away from family to give them a break. Hospital and NDIS working together to help me with transport to Darwin and they are also supporting with respite the same way they help me here at [community] to give my family a rest because they get tired.* (Participant).

These quotes illustrate the primacy of family and community connections in relation to disability, and the need to adequately consider these relationships in the engagement of individuals and in planning processes.

### Engagement with participants

Interviews with participants and support providers highlighted that preparation and explanation of the NDIS and assessment process should occur before initial interactions between people with disability and NDIA staff. They advocated for the initial meetings to be pre-planned or advertised and be conducted with cultural sensitivity and clear communication, especially in the initial stages of participant engagement. A number of participants indicated that they felt apprehensive, confused or scared when people from the NDIS showed up unexpectedly.

*… that time I didn’t know they were coming. I wasn’t expecting them.* (Participant).

*I was confused that time. They just popped up out of nowhere. I didn’t know they were coming. I was surprised and a little bit frightened … When they come I want a straight story from them that I can understand … They missed me with their words they were using. I rang up J....... and asked her who they were. We didn’t know.* (Participant).

Participants who had had undergone planning meetings with NDIA staff without support did not understand what was taking place, as illustrated by the quote below. This quote also highlights the intimate and sensitive nature of some of the conversations undertaken without appropriate prior notice.

*Our participants, not even knowing that they’d had a planning session like or someone came to see me today, and they wanted to know the ins and outs of everything about my life, and I don’t really know what it was …* (Provider).

Interviewees tied the confusion provoked by these unexpected visits to a lack of utilisation of existing relationships. As expounded below, some support providers were diligent in attempting to prepare their participants for the transition to the NDIS. This included explaining the nature of the NDIS and what would be covered in the planning meeting so the participant would be able to properly express their needs and what should be included in their participant plans. However, in the first stages of the roll-out, these relationships between participants and providers were not accounted for, leading to NDIA staff approaching participants without the involvement of existing providers.

*So we also did a lot of work to collaborate with the NDIA on the ground leading up to the roll-out to say that our participants want us to be involved, we’ve done this pre-planning, we’re happy to share the information with you … the NDIA kind of ignored all of that and … started doing plans with participants without us involved. Those participants were confused, they didn’t really understand what was happening or what they were really being asked....* (Provider).

The same provider went on to explain that this situation has improved since the NDIS was initially rolled out in the region. They indicated that subsequent collaboration between the NDIA and existing providers has been strengthened, in part through advocacy on the part of the existing providers.

### Participant understanding of the NDIS

In speaking to participants, it was apparent there was a wide range of understanding regarding the NDIS. While there were some participants who had some limited understanding about the new scheme and why it had been put in place, there were no participants interviewed who fully understood the structure of the NDIS or how it functioned. The quote below is from a participant who demonstrated one of the strongest understandings of the NDIS, but also illustrates the limits of this.

*When I first heard about NDIS I was thinking about organisation like MJD Foundation, because they have a helping law and those Balanda* (non-Aboriginal) *people working for them always help Yolŋu. So, I’m thinking NDIS is like that, everyone wants to help and support for people in their everyday life. But I don’t know what each of those letters N D I S really means.* (Participant).

Participants illustrated better levels of understanding when local providers had previously spoken to them about the scheme. However, even with some level of intervention on the part of providers, this did not always ensure a good understanding for participants.

***Participant:*** *Nah I can’t remember – it’s a lady called ahhh … lives at [remote area].*

***Interviewer:*** *So, she came from the NDIS. Did anybody from anywhere else talk to you about NDIS, anybody from MJD Foundation or anywhere? So, N or C or K or J?*

***Participant:*** *Yes, K.*

***Interviewer:*** *So, K talked to you about the NDIS, she explained that new thing was coming and then that lady came over from [remote area].*

***Participant:*** *Yes.*

***Interviewer:*** *And did they explain in English or in Anindilyakwa.*

***Participant:*** *In English.*

***Interviewer:*** *Do you think you got a clear picture? Do you understand what it is all about?*

***Participant:*** *No – not really.*

Although the role of Community Connectors is to facilitate understanding about the NDIS for participants, Community Connectors also indicated that their own training was lacking.

***Interviewer:*** *Was the training you received sufficient, or somewhat okay?*

***Community Connector:*** *Sort of okay. I talked to the trainers at the course that was at the end of last year (2018) and we all agreed that it was just an introduction, but I said we need more training and information about NDIS, how it works. Not enough information was provided to us.*

Both participants themselves and providers spoke of the ongoing uncertainty participants had regarding the impact of the NDIS on existing payments. In 2013, more than half of the Aboriginal and Torres Strait Islander people in the Northern Territory were on income support [31], and the proportion of Aboriginal and Torres Strait Islander people aged over 15 receiving a government pension or allowance as their main source of income increases with remoteness [32]. Given the reliance of this cohort on welfare payments generally, it is likely that this confusion would have negative implications for engagement with and uptake of the NDIS.

*Yes, now I’m remembering when they came to see me. But I didn’t get much help that time. And they take $85 for food, and they also take another $280 from my pay, but I don’t know what that’s for, why they take that money. That’s my money they are taking, but what for?* (Participant).

*There’s still a misunderstanding that the NDIS is going to affect their carers pension, or their disability pension, or all sorts of things.... It’s still not widely understood that the NDIS can be separate. And also, they won’t get the money … And it has to be known out there that the NDIA is an additional service from your carers pension, or that child’s pension, that disability support. It won’t affect your other payments, but that’s a real fear that... My parents pension, my carers pension, or you know my disability pension can disappear.* (Organisational partner).

Community Connectors identified a lack of information being provided to them regarding the financial workings of the NDIS, which would limit their capacity to ensure that participants also had a functional understanding.

***Interviewer: …*** *and what about the big picture story for money and government? Where the money comes from and what is NT Government part and what is Comm Government part of the money story? How they join those two sources of money together to make NDIS happen?*

***Community Connector:*** *Still waiting to hear more about that part of the NDIS story, because they didn’t talk much about that.*

### Planning meetings

A key component of the planning process is that participants are able to exercise choice over who is involved in the planning meeting. There was agreement across NDIA staff, providers and participants that there was capacity to have family members or supporting individuals from organisations included in planning meetings according to the wants and needs of the participant. Community Connectors were also sometimes involved in this process and provided a bridge between NDIA staff and the participant and family members. Community Connectors were more frequently reported to be involved in the planning process in the Northern Territory than in Queensland.

*A really good thing is like for example, if we’re talking to someone who has loss of hearing and requires an Auslan interpreter, you’ll have the Auslan interpreter with them, you’ll have maybe their guardian, you might have a support worker with them that they’ve asked to come along. Generally, we don’t include providers because we want the participant to have choice and control, but, the participant will tell us if they want them there. So, we honour that. We let them tell us what it is that they want to do part of those conversations. Some conversations might require disability advocates to attend as well.* (NDIA staff).

*[Our] health service is really family orientated where at the approval [of] the patient or participant, we involve families. So that’s where our Community Connectors are really important is [in] that they know the families, because they’ve lived in the community all their life. And they’re able to bring them to the planning meetings rather than an interpreter, and in most of our places English is a third or fourth language. And they have three or four dialects that they speak before even engaging English. So, it’s really important that these Connectors engage with the families and get into the meetings where practical.* (Organisational partner).

Participant comprehension of the role of NDIA staff, the reason for the planning meeting and the connection with the eventual delivery of supports was varied. In speaking to participants about what had happened during their planning meetings, it was clear that there was consistent confusion about what the meeting was about, what was expected from them or expected outcomes from the meeting. As the quote below demonstrates, this confusion tended to persist even when the participant had support during the meeting.

***Interviewer:*** *Do you remember talking to anybody about what you wanted – before you went into that NDIS planning meeting?*

***Participant:*** *No.*

***Interviewer:*** *You don’t remember if you did that?*

***Participant****: No?*

***Interviewer:*** *Did anybody give you any help when you were making that plan with that lady? Anybody sit with you?*

***Participant****: Yeah, my Mum, G.*

***Interviewer:*** *G? And did that help was that good?*

***Participant:*** *No.*

***Interviewer:*** *No? she didn’t help?*

***Participant:*** *No, no, yes she did help.*

***Interviewer:*** *She did help? Did you understand what was happening in that planning meeting?*

***Participant:*** *Not – not really.*

In other cases, participants understood what supports they received, but didn’t connect it to the participant plan or the planning meeting. This stems from participants not understanding the change from previous provision models or comprehending the detail of how the NDIS underpins ongoing services. Some participants did demonstrate understanding of the connection between the planning meeting, the eventual participant plan and outcomes from the plan; however, this was unusual.

***Interviewer:*** *Do you feel like you told her a good strong story?*

***Participant:*** *Yeah.*

***Interviewer:*** *Did you tell her maybe you needed good strong equipment? Wheelchair and shower chair?*

***Participant:*** *Yeah – gestures to wheelchair.*

***Interviewer:*** *And then that plan made that happen?*

***Participant:*** *Yeah.*

The use of interpreters was a key theme in the interviews. English is not the first language for many Aboriginal and Torres Strait Islander people, particularly in remote areas [33]. Interviewees indicated that NDIA guidelines allow for the use of National Accreditation Authority for Translators and Interpreters (NAATI) certified interpreters during participant planning meetings—however, it was difficult to find interpreters who had this qualification and were in appropriate relationships, even if they were otherwise very skilled.

*And the other thing is there’s no way you could expect remote Aboriginal community workers that are very good interpreters and translators because it’d be very hard to expect them to have the national accreditation that the NDIA [are] wanting to have.* (Provider).

*And the interpreters are only able to be funded if they’ve got national accreditation. The issue that we face in the Northern Territory is that it’s very difficult to accredit nationally a lot of the Aboriginal languages …*. *Which then means that you can’t necessarily fund people and the right people, because it’s not just that the person can speak the language. There’s a whole lot of other factors that need to be considered as to whether they’re appropriate to provide that interpretation for that individual.* (Provider).

The following quote illustrates some of the complexity that can present in balancing cultural considerations with the need for a NAATI certified interpreter.

*It is so complicated. One of the most complex planning meetings that we’ve had involved 12 people. We had Aboriginal Interpreter Services (AIS) there, that Aboriginal interpreter services had a poisoned relationship with the participant, but the participant absolutely needed Aboriginal interpreter services. So, we had AIS at one side of the room in a chair facing the wall and the participant at the other side of the room with his family around them so they couldn’t see the AIS interpreter, but they were still able to interpret.* (NDIA staff).

## Discussion

As disability service provision continues to move towards a model of personalisation and individualisation, there is increasing opportunity for alignment in services and processes supporting Indigenous cultural expression and retention. However, despite experiencing higher levels of disability, Indigenous people with disabilities globally are more likely to have unmet need for disability services. This includes lower levels of engagement with disability services and programs [3, 34]. As experiencing and severity of disability is associated with aging [1] and the older (45-plus) Aboriginal and Torres Strait Islander population is increasing [35], the provision of disability services for Aboriginal and Torres Strait individuals, families and communities is likely to be of increasing import. For Indigenous people with disability, the eligibility assessment process has the potential to support empowerment of the individual as well as the wider family and community. This can be done by enabling individuals to situate their needs within their cultural context and in relation to their cultural roles, for example attending ceremony, visiting country or engaging in activities such as hunting and fishing.

Cultural competence as an approach to facilitating effective work to be done in cross-cultural situations has largely focused on racial or ethnic differences. Where cultural competence has examined interactions between providers or health care services and people with disability, this has been removed from intersectionality with other marginalised identities [24]. This paper therefore contributes to the literature in providing an examination of cultural competence in working with Indigenous people with disability. The current research highlighted a number of ways culturally competent communication could be enhanced during the initial NDIS eligibility assessment process. First, a more gradual approach to introducing NDIA staff to individuals with disability; second, ensuring that this initial introduction is facilitated by people the individual has a trusted relationship with, including established service providers, community leaders and Community Connectors; and third, appropriate communication undertaken during planning meetings. This communication should be guided by adherence to cultural norms in the use of interpreters and again supported by trusted individuals such as family members and established service providers.

Interviewees reported that a lack of appropriate preparation, support and engagement in the assessment process led to individuals feeling scared, confused and intruded upon by the sudden appearance of NDIA staff, further magnifying suspicion of unfamiliar disability agencies. Under these circumstances, individuals found it difficult to understand what was required of them during planning meetings and were therefore limited in effectively communicating their needs. Evidence regarding the effectiveness of culturally competent practices indicate the importance of consulting with Indigenous health services and communities and ensuring that service delivery is tailored to the community context [21]. While NDIA staff indicated that an engagement process had been followed within communities, there may be a need for more intensive and extensive local engagement, including development of appropriate communication resources, longer periods of active engagement and/or changes in the engagement approach. More robust engagement will require incorporating the expertise of the Aboriginal community-controlled sector and supporting cultural competency training in a systemic way.

Established relationships resulting in trust, particularly the trust between participants and some support providers, was an important theme in the current research. With the shift towards improving functioning and needs-based and personalised disability services, eligibility assessment has placed a greater emphasis on the articulated needs of service users. Assessment within the framework of personalised care is therefore a collaborative and dynamic process between the individual, the assessor, and any supporting parties [36]. In some instances, participants may want providers that they have an existing relationship with to ‘speak for them’ because of the trust that has been established. The support of existing providers could also be helpful for staff conducting assessments in allowing for difficult topics to be broached sensitively and appropriately . In one study on the assessment process in personalised care, practitioners expressed a certain reticence to approach what they considered to be overly ‘intrusive’ or ‘sensitive’ topics, which could ultimately have a negative impact for the individual’s allotment [36]. In such cases, the presence of a service provider who is already trusted by the individual and able to support accurate responses to such topics could mitigate this impact. However, providers are normally discouraged from attending planning meetings in order to prevent what may be perceived as advocacy on the part of providers and to ensure participants are able to freely choose providers. While processes to mitigate potential conflicts of interests are needed, this should be balanced with the empowerment of the individual to clearly express their identified needs. This would mean instituting accountability mechanisms directly with the support provider, rather than during the participant interaction or planning processes.

Given that English is not a first language for many Aboriginal and Torres Strait Islander people, particularly in remote areas [33], and the low level of understanding participants had of the scheme, it is apparent that appropriate use of interpreters is crucial to ensuring effective and comprehensible communication. However, interviewees reported that interpreters were used in ways that contradicted cultural norms regarding kinship and cultural relationships. Having access not only to an interpreter but to the correct interpreter, taking into account kinship and cultural relationships, is essential to providing culturally appropriate planning processes. While using a certified interpreter is espoused to be the first choice in meeting with participants who do not have English as a first language, where this cannot accommodate important social/cultural mores then other options (e.g., use of Community Connectors as language and cultural ‘brokers’) should be considered. This is particularly important if the only alternative is delays in implementation or the use of culturally inappropriate interpreters. Models for funding and supporting Community Connectors to fulfil this role or facilitate others to do so should be explored.

In order for assessment and planning to be undertaken in a culturally-appropriate and holistic manner, these processes should incorporate Indigenous perspectives of health and disability, cultural practices (such as storytelling) and familial and communal roles of caring [18, 37, 38]. In the current study, participants’ experiences of disability reflect the centrality of family. In viewing a cultural competence framework through a disability lens, Eddey and Robey (2005) highlight the care load on family members as an important factor in how individuals and families view disability and the resulting perception of disability support needs [23]. As kinship is an important element of Indigenous family structures and way of life, caring for individuals with a disability is often considered to be a responsibility of family members, rather than external support workers or support providers [16, 39]. Family support during the planning process was therefore seen as essential. However, effective family support of participants during the planning process requires additional explanatory resources. Planning meetings may need to be contextualised and further structure provided as well as prioritising the development of resources to guide these conversations. The development of culturally safe and appropriate resources should involve local co-design to ensure that they reflect the diversity of Indigenous communities and are locally applicable.

Table 2 outlines the elements of culturally competent communication identified in the current research and recommended approaches to addressing the same.

**Table 2:** Elements of culturally competent communication in Indigenous disability assessment and recommended strategies

Element of culturally competent communication	Recommended strategies to promote culturally competent communication in disability assessment
A supported approach to introducing unfamiliar staff members to individuals with disability	● Full implementation of the NDIA Aboriginal and Torres Strait Islander Engagement Strategy ● Build on engagement at higher institutional levels to support engagement with communities, families and individuals ● Extensive and intensive local engagement with the broader community, community leaders and the Aboriginal community-controlled health sector ● Gradual introduction of unfamiliar staff members ● Adequate and appropriate cultural competence training for staff members systematically provided
Initial introduction is facilitated by people the individual has a trusted relationship with	● Engagement is supported and facilitated by existing providers ● Funding and support for Community Connectors to function as language and cultural ‘brokers’
Appropriate communication undertaken during planning meetings	● Interpreter support for individuals and families that do not have English as a first language ● Use of interpreters that adheres to cultural needs and norms, such as kinship and cultural relationships ● Protocols are established to enable the participation of existing providers in assessment processes while managing potential conflicts of interest ● Families are provided with sufficient explanation and context to be able to meaningfully participate ● Use of cultural practices such as storytelling in assessment and planning meetings ● Individuals’ needs situated within their cultural context and roles ● Communication resources that are place-based and culturally safe developed through co-design

### Strengths and limitations

This article provides an examination of cultural competence in working with Indigenous people with disability, thus responding to a gap between the literature on inter-racial/inter-ethnic cultural competence and cultural competence in providing disability support services. The research was strengthened by the input and involvement of Aboriginal and Torres Strait Islander people at all stages. In particular, the partnerships with MJDF and Synapse were crucial to the project at all stages, from research design to data collection and analysis. The involvement of the two organisations provided an essential perspective of individuals who had trusted relationships with Aboriginal and Torres Strait Islander people with disability as long-standing providers. Along with the PRG, they could therefore provide additional context and insight into the analysis that would not have been available otherwise. The study is limited in scope, as participants were recruited from remote areas of two Australian jurisdictions; its findings may therefore not be widely generalisable. However, findings from the study concur with established principles of cultural competence.

## Conclusions

Indigenous people exhibit lower levels of engagement with disability services than their non-Indigenous counterparts, despite experiencing a higher burden of disability. While the principles of cultural competence have been utilised to address racial and ethnic inequities in health, cultural competence frameworks have more rarely been applied to disability services and intersectionality between the two is nearly wholly lacking. Interviews indicated that a lack of cultural competence on the part of NDIA staff negatively impacted on individuals’ understanding of the NDIS and therefore capacity to effectively advocate for their needs. Cultural competence principles should be applied in order to ensure that disability assessment and planning reflects the needs of Indigenous individuals with disability and their families. Cultural competence in disability assessment and planning can be strengthened through multi-level engagement with the Aboriginal community-controlled sector and community leaders. Implementing mechanisms to enable the involvement of families, trusted service providers and Community Connectors can support a more meaningful understanding of individuals’ needs within their cultural context and in relation to their cultural roles.

## Supplementary Information

**Additional file 1.** Supplementary material: Interview schedules.

## Data Availability

The datasets generated and/or analysed during the current study are not publicly available to protect the confidentiality of individuals, but are available from the corresponding author on reasonable request.

## Abbreviations

AIS: Aboriginal Interpreter Services
FPDN: First Peoples Disability Network Australia
HREC: Human Research Ethics Committee
ICF: International Classification of Functioning, Disability and Health
MJD: Machado-Joseph disease
MJDF: MJD Foundation
NAATI: National Accreditation Authority for Translators and Interpreters
NDIA: National Disability Insurance Agency
NDIS: National Disability Insurance Scheme
PRG: Project Reference Group
WHO: World Health Organization
