# Supplementary material for: Culturally competent communication in Indigenous disability assessment: a qualitative study
Source: Int J Equity Health. 2021 Mar 1;20:68. doi: 10.1186/s12939-021-01402-9 (PMC7923453; doi:10.1186/s12939-021-01402-9)
Supplement: Supplementary file 1 — Additional file 1. Supplementary material: Interview schedules. [file 12939_2021_1402_MOESM1_ESM.docx]

## Supplementary material: Interview schedules

### Participant interview schedule

**Is the National Disability Insurance Scheme meeting the needs of Aboriginal and Torres Strait Islander people? Evaluating the roll-out in Queensland and the Northern Territory.**

**Participant Interview protocol**

The interviewer will begin by discussing the purpose of the research study. She (he) will talk through the ‘plain language statement’ and make sure the NDIS participant has given informed consent about participating. She (he) will also check verbal consent has been given to allow audio recording of the interview.

**Demographics**

*To start off let’s talk a little bit about your background and family.* (*nb*: answers to structured questions will be recorded electronically on a separate response sheet)

1. Where do you live?
2. Which communities or homelands do you often visit? What clan and language groups do you belong to?
3. How old are you? (Age in years)
4. Do you have a partner? Are you married? Yes/No
5. Do you have any children? If so, what ages are your children? (Age in years each child) 6. Are you working at the moment? (Full-time/part-time /unemployed) 7. How far did you go at school?

a. Did you study after school?

8. Do you have a health care card? (Yes/No)

**Disability**

*Now I am going to ask some questions about your disability and your everyday life.*

1. Tell me a little bit about your disability.
2. How is your daily life affected? (i.e., participation with family, body movement and other health impacts)?
3. Do you have any health conditions that your doctor/nurse knows about? Does this affect your disability?
4. Do you have someone who cares for you, helps you with your life? Who is the person who helps you the most?
   1. Who is your number one carer?
   2. What things do you need special help with?
5. What special help do you receive?

i. What was your part in getting the kinds of help you are receiving?

1. What are the main things where you would like extra support? What changes do you want to see with the support you are receiving now?
2. Over the last full year (12 months) do you feel that you have been treated unfairly by others? Have you experienced other problems? Where did this happen?
   1. Why do you think they treated you this way (i.e., because you are disabled, Aboriginal/other)?
   2. What effect did this have on you?

**Initial engagement with the NDIS**

*Starting up the NDIS has changed the way disability services are provided. I want to ask you some questions about what you’ve heard and understand about the NDIS.*

1. Have you heard about the NDIS? Do you know what the NDIS is?
2. Who first told you about the NDIS? When?
3. Did they explain about NDIS to you in your own language? Did what they say give you a clear picture about the NDIS? Tell me what you understand about the reason for NDIS and what it is aiming to do.
4. Did you think it would be good to “sign up” for the NDIS?
5. Did you complete an Access Request Form (ARF) to access the NDIS? Did you have help to complete the ARF to access the NDIS?
6. Did you understand how to complete the ARF? Did you understand the questions?
7. How was your ARF sent to the NDIS?
8. Have you heard back from the NDIS? Did the NDIS get back in touch with you?
   1. Was your ARF accepted?
   2. Was your ARF rejected? If so, did you know you could appeal the NDIA decision? Did you appeal? Was your appeal successful?
9. Are you a participant in NDIS (National Disability Insurance Scheme)? If yes, for how long?

| **If a participant with NDIS (i.e., you have a NDIS Plan), continue** |  |
| --- | --- |
| **If not a client of the NDIS, go to ‘National Disability Insurance Scheme (Non-clients)** | |

**National Disability Insurance Scheme**

*I’d like to ask you some questions about how some things may have changed since you’ve been on the NDIS.*

1. Why did you decide to start using NDIS? Can you tell me what you thought about how NDIS might help you?
2. Have you noticed changes in the care/support you receive since you got an NDIS plan?
   1. How have these changes affected the way you are cared for?
   2. Have these changes made your life better?
   3. Have there been some changes that were not good?
   4. What things have helped make your care better? What are some of the problems that stop you receiving good care?

**Planning**

*All people in NDIS have a Plan for their care. Each person’s approved Plan is like the ‘law’ (or rules) for how that person with a disability receives help for their disability. Let’s talk together about the way plans were made for your care.*

1. When did you first start talking about having a Plan for NDIS help?
2. Did anyone talk to you about the steps involved in making a Participant Plan? Who talked to you and when? Were you given any help to get ready for making your Plan? If Yes, how did this information help you with the different planning steps?
3. What were you thinking would happen in the planning meetings?
4. Where did the planning meetings take place? Was it face-to-face or over the phone? Was it a good way to talk about Plans?
   1. Did you understand what happened in those meetings?
5. How do you feel about the way your Plan was made?
6. Do you think making Plans should be done a different way?
7. What do you hope comes from your Plan? What do you want to see happen?
8. Who was the main person in charge of the planning?
   1. Do you think this person was the right person to be in charge? If Yes, why? If No, why not? Who might have done a better job?
9. Were there other people you wanted to talk in the planning meeting (e.g., your family)? Were you able to talk with them or ask them about helping with planning?
   - 1. If No, what made it hard?
     2. If Yes, did they like being in the planning meetings?
10. Who helped you talk about the things you would like to see in your Plan? What made it easy to talk about making a Plan? What made it hard to talk about making your Plan?
11. Do you think other people involved really understood what you were saying and feeling, or only a little bit?
12. What things in your Plan make you feel good about being a Warnindilyakwa / Yolŋu/Yugul Mangi person? Do you want more activities from your culture to be included? What types of activities do you enjoy doing?
13. Does your NDIS Plan fit with the things you need help with? If No, what is the reason for that?
14. What do you think you can do to change what is in your Plan?
15. What things about the visit by NDIA staff were helpful and made you feel like they understood how you felt as a Warnindilyakwa/Yolŋu/Yugul Mangi person? What things about their visit were not helpful?
16. What would you like to see happen to make future planning more easy?

**Implementation**

*Now let’s talk about what care and support you are receiving.*

1. Do you feel you now have more choice in the care and support you receive? Do you like being in the NDIS?
2. What were the main points you wanted to see in your Plan?
3. Have any Warnindilyakwa / Yolŋu/Yugul Mangi culture activities been part of your care? Do you think they should be?
4. How much care/support that is written in your Plan do you receive right now? What helped? What things stopped good care from happening?
5. Who are the different people or organisations you go to for your care or support? Is there someone else you would like to see to help with your care? Is there anyone you currently see who you are unhappy with?
6. What care/support is working well for you That you are happy about? What are your main concerns about the care/support you are receiving?
7. What things about your care helped you feel good as a Warnindilyakwa/Yolŋu/Yugul Mangi person?
8. In what ways has your care become better? What are main things you would like to see for a better future?

**If a client of the NDIS, go to ‘Other services’**

**National Disability Insurance Scheme (Non-clients)**

*Starting up the NDIS has changed the way disability services are provided.*

1. Why did you decide not to use NDIS?
2. Do you think you might like to join with NDIS in the future?

a. What is your reason for saying that?

1. Starting with NDIS is sometimes a bit difficult. Are you interested in finding out some more about NDIS in your first language?

**Other services**

*People with disabilities use a range of services (e.g., health, social services).*

1. What types of disability help are there for you to use?
2. How well do different services work together to give you good care? How could this be done better?

**Overall**

1. How could disability care and support be done better so Aboriginal and Torres Strait Islander people feel they are being cared for properly by the NDIS?
2. Is there anything else you would like to talk about?

Thank you for doing this interview and being part of our research project.

### NDIA staff interview schedule

**Is the NDIS (National Disability Insurance Scheme) helping Aboriginal and Torres Strait Islander people the right way? How do people feel**

**about the way NDIS started in the Northern Territory and Queensland?**

**Guidelines for doing Interviews**

In this interview we will discuss the roll out of NDIS (National Disability Insurance Scheme) in the NT/QLD.

We are really interested in the main points learnt along the way and also how things are working now.

We will use an interpreter/language helper to assist people who do not use English as their first language.

**Background**

*Let’s start off talking a little bit about your history of working with NDIS.*

1. What is the name of your work position?
2. Who do you work for?
3. Are you Aboriginal or Torres Strait Islander?
4. What is your role with NDIS?
5. How long have you been doing NDIS work?
6. What do you understand is the main reason for NDIS?
7. What are the barriers and facilitators to NDIA staff delivering this?
8. What do you know of the NDIS’s Aboriginal and Torres Strait Islander Engagement Strategy? How does it inform you in your daily work?
9. What activities to you undertake within Aboriginal and Torres Strait Islander communities to promote access to the Scheme?

**Signing up providers**

*One the first steps to implementing the NDIS was signing up providers.*

1. Can you tell me about the process of signing up providers? What worked well? What could have been improved?
2. How satisfied are you with the number and diversity of providers who signed up?
   1. Where did the group of the providers need strengthening?
   2. Were any strategies implemented to support this?
3. For service providers that have signed up with the NDIS:
   1. What level of experience do they have in working with Aboriginal and Torres Strait Islander people and communities?
   2. For those providers that did not have much experience in this area or were hesitant, was there any support provided? If so, what kind of support and if not, what type of support might have been useful?
4. What are levels of participation like among providers who work specifically with Aboriginal and Torres Strait Islander people?
   1. Were there any strategies implemented to support greater participation among these providers?
5. How much choice do people with disabilities, especially Aboriginal and Torres Strait Islander people, have in their care/support given the range of participating providers?
   1. Is there any evidence of an emerging market?
6. What are the main learnings from the initial sign-up process?

**Recruiting and training the workforce**

*The NDIS also required the recruitment and training of a new workforce. Now I would like to talk a bit about that process.*

1. In what ways did the recruitment of NDIA staff in NT/QLD work well? What were the challenges?

1. Were there local applicants? What was the representation of diverse populations particularly Aboriginal and Torres Strait Islander people like?
   1. What strategies were implemented to address any issues with recruitment?
2. What were the strengths and weaknesses in the training NDIA staff?
   1. Have any strategies been put in place to improve training?
3. What provision was made to adapt training and recruitment to local context?
4. How were cultural concepts and understanding of disability and cultural safety addressed in training?
5. What were the key priorities of NDIA staff in terms of managing clients?
6. How could recruitment and training of staff be improved in the future?

**Preparing for the roll-out**

*Now I would like to talk about what preparation there was for the roll-out of the NDIS in NT/QLD in terms of engaging with local service providers, Aboriginal and Torres Strait Islander Community Controlled organisations and Aboriginal and Torres Strait Islander communities*

1. What engagement was there with local service providers leading up to the roll-out of the NDIS? What worked well? What could have been improved?
2. What engagement was there with Aboriginal and Torres Strait Islander Community Controlled organisations and Aboriginal and Torres Strait Islander communities leading up to the roll-out of the NDIS? What worked well? What could have been improved?
3. How was engagement with Aboriginal and Torres Strait Islander communities undertaken? How and who decided which community members should be engaged with?
4. Have you worked with Aboriginal and Torres Strait Islander community connectors?
   1. How do you see their role?
   2. How have you worked with them?
   3. Have they been effective in building connections? How could this effectiveness be improved?
5. Has there been differences in how much services engage with the NDIS? In what way has this manifested? What drives these differences?
6. What are the main learnings from the engagement process?

**Overall**

1. What are the main things we can learn about how to do NDIS work better?
2. Are there any other things you would like to discuss?

Thank you for your time.

### Partner organisations/ Community Connectors interview schedule

**Is the NDIS (National Disability Insurance Scheme) helping Aboriginal and Torres Strait Islander people the right way? How do people feel**

**about the way NDIS started in the Northern Territory and Queensland?**

**Guidelines for doing Interviews**

In this interview we will discuss the roll out of NDIS (National Disability Insurance Scheme) in the NT/QLD. We are really interested in the main points learnt along the way and also how things are working now.

**Background**

*Let’s start off talking a little bit about your history of working with NDIS.*

1. What is the name of your work position?
2. Who do you work for?
3. Are you Aboriginal or Torres Strait Islander?
4. What is your role with NDIS?
5. How long have you been doing NDIS work?
6. What do you understand is the main reason for NDIS?
7. What are the barriers and facilitators to NDIA staff delivering this?
8. What do you know of the NDIS’s Aboriginal and Torres Strait Islander Engagement Strategy? How does it inform you in your daily work?
9. What activities to you undertake within Aboriginal and Torres Strait Islander communities to promote access to the Scheme?

| →**Providers continue** |  |
| --- | --- |
| →**Community Connectors go to ‘Community connectors’** | |

**Implementing planning for clients**

*A key component of the NDIS is the development of individual plans to ensure that services better meet the needs of people with disabilities. I am going to ask some questions about the implementation of the planning process in NT/QLD*

1. To what extent was the planning process implemented as planned? What worked well? What were some of the challenges?
2. Have any changes been made to the planning processes or their support in response to these learnings?
3. Have there been differences in how planning has been done in different communities?
4. What evidence is there that the planning process was conducted in culturally safe ways for Aboriginal and Torres Strait Islander people?
   1. What are areas for improvement?
5. What scope is there for families to be included in the planning process?
   1. How is this implemented?
6. What scope is there for cultural activities to be included in client plans? Does this vary according to who involved in the planning e.g., NDIS staff or LAC’s or other agency/family or by geography (urban vs rural vs remote areas)?
   1. What are some instances where planning may include cultural activities?
   2. What types of activities may be included?

1. Is there scope for other organisations to be involved in the planning process (e.g., other services that may be providing care to the client)?
2. What are the main learnings from the implementation of the planning process?

**Providing services**

*Now I am going to ask you about the provision of services though the NDIS in NT/QLD*

1. To what extent have people with disabilities been able to receive the care/support outlined in their plans? What are the barriers and facilitating factors?
2. To what extent have Aboriginal and Torres Strait Islander people with disabilities been able to receive the care/support outlined in their plans? What are the barriers and facilitating factors?
3. How is the NDIA working with service providers to ensure that appropriate care/support is provided?
   1. What policies are in place for ongoing communication with services?
4. What evidence is there that the care/support people are being provided under the NDIS is better meeting their needs than previous arrangements? What areas for improvement are there?
5. What do you consider to be culturally safe care for Aboriginal and Torres Strait Islander people and communities?
   1. What evidence is there that services are being provided in a culturally safe way for Aboriginal and Torres Strait Islander people?
   2. What are areas for improvement?
   3. What support is available for providers that struggle to provide culturally safe care?
6. Have there been differences in the way services are provided in different communities?
7. What are the main learnings in terms of service provision?

→**Providers go to ‘Overall’**

**Community Connectors**

• **Recruiting and training**

*I’d like to talk to you about the first steps of how you started working with NDIS. What is your role with NDIS?*

1. Can you tell me how you first heard about NDIS? How was your job as a Community Connector explained to you?
2. What are the communities where you did work with the NDIS?
3. What training was provided to you when you began your NDIS role? Did anyone talk with you about ‘cultural safety’ or feeling comfortable about your NDIS work?
4. Did you receive the training in your first language?
   1. How did you feel about the training you had?
   2. How do you think the training could be made better in future?
5. Has your role with NDIS been different from what you expected? In what way was it different?
6. Did you work in more than one community?
   1. How is the work different in different places?
   2. Do you feel you could change the way you talked about NDIS to help people better understand?

• **Preparing for the roll-out**

*Now I would like to talk about your job in preparing for the start-up of NDIS in NT/QLD and the way you worked with local service providers, and local organisations.*

1. How did you help with getting ready for the NDIS to start in the communities you worked in? Did you work with local service providers, and local organisations?
   1. How did you help people get ready for the NDIS to start?
   2. Did you work with local service providers and local organisations?
2. What meetings happened with Aboriginal and Torres Strait Islander people and Aboriginal and Torres Strait Islander organisations before the NDIS started? What worked well? What might have been done better?
3. How did discussions with Aboriginal and Torres Strait Islander communities happen?
   1. Who decided which community leaders to talk with?
4. Has there been much interest in NDIS?
   1. Have disability people received more help since NDIS started?
   2. Have other people also received more help since NDIS started
   3. What extra help have you seen?
   4. What do you think are reasons for some things being better or different?
5. What are the main things you have learnt from helping NDIS to start?

• **Implementing planning for clients**

*For the NDIS to happen, each disabled person has their own personal Plan to say what help they can have and who will give that help. I am going to ask you some questions about what you did when Plans were being made.*

1. What was your role/job in helping people to have Plans?
2. What worked well? What were some of the challenges/problems?
3. What strategies has the NDIA been implemented to improve planning?
   1. Can you think of some ways? (e.g., family being involved, cultural help)
4. Do you think the way plans were done helped Aboriginal and Torres Strait Islander people to feel comfortable?
   1. What are the best ways to make plans?
5. Tell me about the way you work with families to do plans?
6. How are Aboriginal and Torres Strait Islander ways included in the plans of people who have a disability
   1. What types of activities things do people want to see included?
   2. What are some good ways to include more Aboriginal and Torres Strait Islander ways of doing things in Plans?

**Overall**

1. What are the main things we can learn about how to do NDIS work better?
2. Are there any other things you would like to discuss?

Thank you for your time.
